# 2D and 3D Angles-Only Target Tracking Based on Maximum Correntropy Kalman Filters

**DOI:** 10.3390/s22155625

**Published:** 2022-07-27

**Authors:** Asfia Urooj, Aastha Dak, Branko Ristic, Rahul Radhakrishnan

**Affiliations:** 1 Department of Electrical Engineering, Sardar Vallabhbhai National Institute of Technology, Surat 395007, India; ds19el006@eed.svnit.ac.in (A.U.); p20ic001@eed.svnit.ac.in (A.D.); r.rahul@eed.svnit.ac.in (R.R.); 2 School of Engineering, RMIT University, Melbourne, VIC 3000, Australia

**Keywords:** nonlinear filtering, non Gaussian noise, maximum correntropy criterion, Gaussian kernel, Cauchy kernel

## Abstract

In this paper, angles-only target tracking (AoT) problem is investigated in the non Gaussian environment. Since the conventional minimum mean square error criterion based estimators tend to give poor accuracy in the presence of large outliers or impulsive noises in measurement, a maximum correntropy criterion (MCC) based framework is presented. Accordingly, three new estimation algorithms are developed for AoT problems based on the conventional sigma point filters, termed as MC-UKF-CK, MC-NSKF-GK and MC-NSKF-CK. Here MC-NSKF-GK represents the maximum correntropy new sigma point Kalman filter realized using Gaussian kernel and MC-NSKF-CK represents realization using Cauchy kernel. Similarly, based on the unscented Kalman filter, MC-UKF-CK has been developed. The performance of all these estimators is evaluated in terms of root-mean-square error (RMSE) in position and % track loss. The simulations were carried out for 2D as well as 3D AoT scenarios and it was inferred that, the developed algorithms performed with improved estimation accuracy than the conventional ones, in the presence of non Gaussian measurement noise.

## 1. Introduction

In state estimation, Kalman filter (KF) is a recursive solution used in various applications, such as information fusion, system control, integrated navigation, target tracking, and GPS solutions [1,2,3,4]. Kalman filter gives optimal estimates provided the dynamical system is linear and the noises assumed are Gaussian. However, it is extended to nonlinear systems through suitable approximation of the nonlinear functions. Using the Taylor series to linearize the nonlinear functions, the popular extended Kalman filter (EKF) [5] was derived. Also various sigma point filters have been proposed in the literature such as unscented Kalman filter (UKF) [6], cubature Kalman filter (CKF) [7], new sigma point Kalman filter (NSKF) [8], to obtain improved estimation accuracy than the EKF.

Since these filters are based on minimum mean square error criterion, their performance is likely to get deteriorated in the presence of non Gaussian noises such as heavy tailed and impulsive noises [9]. This makes state estimation a very challenging problem in the presence of nonlinear models and non Gaussian noise. Other possible solutions that can provide robust state estimates are Gaussian sum filter (GSF) [10,11], particle filter (PF) [12], Huber’s KF (HKF, also known as M-estimation) [13], $H_{\infty }$ filter [14] etc.

In order to improve the robustness of nonlinear state estimators in the presence of non Gaussian noise, a local similarity measure called correntropy [15,16], based filter called correntropy filter (C-Filter) was first proposed in [17]. Since it was developed by replacing the minimum mean square error (MMSE) criterion with maximum correntropy criterion (MCC), it proved beneficial for non Gaussian systems, but only for linear systems [18]. This algorithm made use of least squares and fixed point iteration, but failed to incorporate covariance estimation. In order to avoid this, a maximum correntropy Kalman filter (MCKF) involving fixed point iteration and covariance propagation was proposed in [19]. Similar issue was also addressed in [20], which used a cost function consisting of weighted least square (WLS) and Gaussian kernel function, and hence was named as maximum correntropy criterion-Kalman filter (MCC-KF).

To deal with nonlinear systems, extensions to the existing conventional algorithms based on MCC criterion were also developed and were named as maximum correntropy EKF (MC-EKF) [21], maximum correntropy UKF (MC-UKF) [22] and maximum correntropy sparse grid Gauss-Hermite quadrature filter (MC-SGHQF) [23]. But in the presence of large outliers in measurements, these filters incurred analytical problems in calculating inverse of matrices. Thus, new algorithms involving new cost function, WLS and statistical linearisation were proposed in [24], which were called as MC-UKF-constant and MC-UKF-adaptive [25]. In developing the above mentioned estimators, Gaussian kernel played an important role in suppressing the non Gaussian measurement noise. In target tracking applications, we may receive measurements which have larger outliers. This could prove to be a challenging task in successful estimation of states using Gaussian kernel as it may be difficult to select a proper kernel bandwidth. Hence, Gaussian kernel may not always prove to be the best choice for a kernel function. To overcome this drawback, a Cauchy kernel function is constructed which gives reasonable estimation accuracy for a wide range of kernel bandwidth [26,27].

This paper deals with angles only target tracking problem in 2D and 3D. The literature presents with many variations of this tracking problem such as when the target is a curvilinear manoeuvring target [28,29]. However, as is common in passive sonar target tracking applications, the objective here is to estimate the states of a moving constant velocity target with the help of angles-only measurements, but corrupted with non Gaussian noise. The observer continuously monitors for the signals, that are generated due to the sound radiated by the target. The AoT can also be performed with other measurement sources like IRST sensor [4], radar [30] and also through video tracking [31]. Any irregularities in these signals received by the observer can be considered as glint noise. A mixture model of two zero-mean Gaussian for glint noise has been proposed in [32]. This consists of one Gaussian density with high probability and small variance while the other has small probability of occurrence and large variance. Alternatively, it is also modelled in [33] as a mixture of zero mean with small variance. In this work, the non Gaussian noise in angular measurements is modelled as a mixture of Gaussian densities plus shot noise.

The main contribution of this paper is the development of three new nonlinear filters for AoT problem, MC-UKF-CK, MC-NSKF-GK and MC-NSKF-CK, and their performance evaluation in the context of angles-only tracking. Accordingly, conventional filters UKF and NSKF have been reformulated based on maximum correntropy criterion. MC-UKF and MC-NSKF based on Gaussian kernel (MC-UKF-GK, MC-NSKF-GK) and Cauchy kernel (MC-UKF-CK, MC-NSKF-CK) functions have been derived. The performance evaluation of these estimators are conducted considering RMSE in position and track loss as the two performance metrics and a comparative discussion is presented. The simulation results highlight that the existing solutions behave poorly in comparison to the proposed algorithms.

The rest of the paper is organised as follows. Section 2 describes the problem formulation for AoT in 2D as well as 3D. Section 3 illustrates the correntropy, its properties for two random variables and MCC. In Section 4, the already existing Gaussian kernel based MC state estimation framework is revisited. In Section 5, the Cauchy kernel based MC state estimation framework for nonlinear systems is derived. Section 6 briefly discuss about the state estimators on which the developed MCC framework is incorporated. Section 7 describes the realization of non Gaussian noise, followed by simulation study in Section 8. Finally, the concluding remarks are given in Section 9.

## 2. Problem Formulation

The aim of the angles only tracking problem is to track the target trajectory using the noise corrupted angular measurements. The dynamics of the target is assumed to be a constant velocity motion. The observer motion is deterministic, implying that the position and velocity of the observer is known to us. The 2D and 3D target observer dynamics is illustrated below.

### 2.1. Process Model

The target and observer state vector with position and velocity as its states is given as

$$\begin{array}{cccc} X_{k}^{t} & ={\left[\begin{array}{cccc} x_{k}^{t} & y_{k}^{t} & {\dot{x}}_{k}^{t} & {\dot{y}}_{k}^{t} \end{array}\right]}^{\prime } & X_{k}^{o} & ={\left[\begin{array}{cccc} x_{k}^{o} & y_{k}^{o} & {\dot{x}}_{k}^{o} & {\dot{y}}_{k}^{o} \end{array}\right]}^{\prime }. \end{array}$$

The discrete time linear process model representing the target motion is given as

(1) $$X_{k}^{t}=FX_{k-1}^{t}+w_{k-1}.$$

Now, the relative state vector dynamics is

(2) $$X_{k}=FX_{k-1}+w_{k-1}-X_{k-1}^{o}+FX_{k-1}^{o}.$$

where $X_{k}$, the relative vector is defined as

(3) $$\begin{array}{cc} X_{k} & =X_{k}^{t}-X_{k}^{o}\\ \; & ={\left[\begin{array}{cccc} x_{k}^{t}-x_{k}^{o} & y_{k}^{t}-y_{k}^{o}\; & {\dot{x}}_{k}^{t}-{\dot{x}}_{k}^{o}\; & {\dot{y}}_{k}^{t}-{\dot{y}}_{k}^{o} \end{array}\right]}^{\prime }\\ \; & ={\left[\begin{array}{cccc} x_{k} & y_{k} & \dot{x_{k}} & \dot{y_{k}} \end{array}\right]}^{\prime }. \end{array}$$

F is the state transition matrix and $w_{k-1}$ is zero mean Gaussian process noise with Q as the covariance matrix. For problem formulation in the two dimensional space (let *n* = 2), F, Q matrices are defined as,

$$\begin{array}{cccc} F & =\left[\begin{array}{cccc} 1 & 0 & T & 0\\ 0 & 1 & 0 & T\\ 0 & 0 & 1 & 0\\ 0 & 0 & 0 & 1 \end{array}\right] & \mathrm{and}\;Q & =\left[\begin{array}{cccc} \frac{T^{3}}{3}q_{x} & 0 & \frac{T^{2}}{2}q_{x} & 0\\ 0 & \frac{T^{3}}{3}q_{y} & 0 & \frac{T^{2}}{2}q_{y}\\ \frac{T^{2}}{2}q_{x} & 0 & Tq_{x} & 0\\ 0 & \frac{T^{2}}{2}q_{y} & 0 & Tq_{y} \end{array}\right]. \end{array}$$

**Figure 1:**
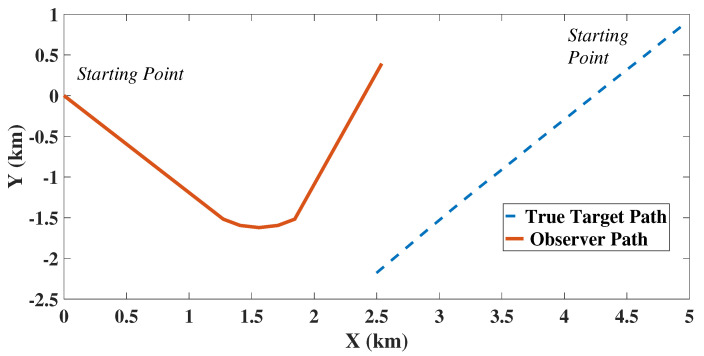
Target Observer Dynamics in 2D.

The target observer dynamics in 2D for a moderately nonlinear scenario, is shown in Figure 1. Similarly for *n* = 3, the state and the associated matrices are

$$\begin{array}{cccc} {X^{t}}_{k} & ={\left[\begin{array}{cccccc} x_{k}^{t} & y_{k}^{t} & z_{k}^{t} & {\dot{x}}_{k}^{t} & {\dot{y}}_{k}^{t} & {\dot{z}}_{k}^{t} \end{array}\right]}^{\prime } & X^{o} & ={\left[\begin{array}{cccccc} x_{k}^{o} & y_{k}^{o} & z_{k}^{o} & {\dot{x}}_{k}^{o} & {\dot{y}}_{k}^{o} & {\dot{z}}_{k}^{o} \end{array}\right]}^{\prime } \end{array}$$

$$\begin{array}{cccc} F_{k-1} & =\left[\begin{array}{cccccc} 1 & 0 & 0 & T & 0 & 0\\ 0 & 1 & 0 & 0 & T & 0\\ 0 & 0 & 1 & 0 & 0 & T\\ 0 & 0 & 0 & 1 & 0 & 0\\ 0 & 0 & 0 & 0 & 1 & 0\\ 0 & 0 & 0 & 0 & 0 & 1 \end{array}\right], & Q_{k-1} & =\left[\begin{array}{cccccc} \frac{T^{3}}{3}q_{x} & 0 & 0 & \frac{T^{2}}{2}q_{x} & 0 & 0\\ 0 & \frac{T^{3}}{3}q_{y} & 0 & 0 & \frac{T^{2}}{2}q_{y} & 0\\ 0 & 0 & \frac{T^{3}}{3}q_{z} & 0 & 0 & \frac{T^{2}}{2}q_{z}\\ \frac{T^{2}}{2}q_{x} & 0 & 0 & Tq_{x} & 0 & 0\\ 0 & \frac{T^{2}}{2}q_{y} & 0 & 0 & Tq_{y} & 0\\ 0 & 0 & \frac{T^{2}}{2}q_{z} & 0 & 0 & Tq_{z} \end{array}\right], \end{array}$$

where *T* is the sampling time interval and $q_{x},q_{y},q_{z}$ are the power spectral densities of the process noise along the *X*, *Y*, and *Z* axes respectively.

**Figure 2:**
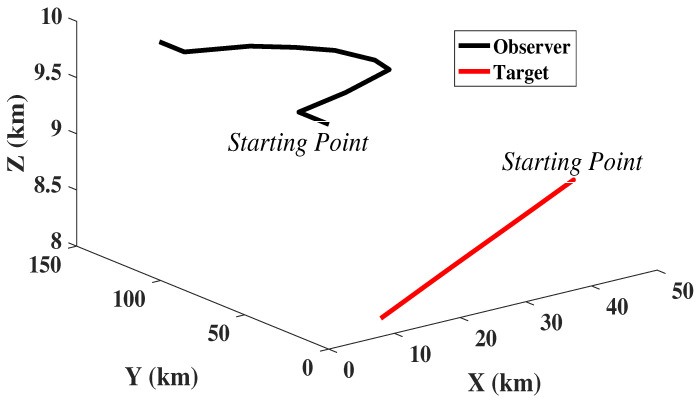
Target Observer Dynamics in 3D.

The 3D target observer trajectory referred in the problem is given by Figure 2.

### 2.2. Measurement Model

**2D AoT problem:** The only available measurements are the bearing angles through which the states of the relative state vector can be estimated. The measurement model and the true angle measurements for the problem can be represented as

(4) $$\begin{array}{cccc} z_{k} & =h\left(X_{k}\right)+v_{k} & h\left(X_{k}\right)=\beta_{k} & =\tan^{-1}\left(x_{k},y_{k}\right). \end{array}$$

where $v_{k}$ shall be modelled as the non Gaussian noise.

**Figure 3:**
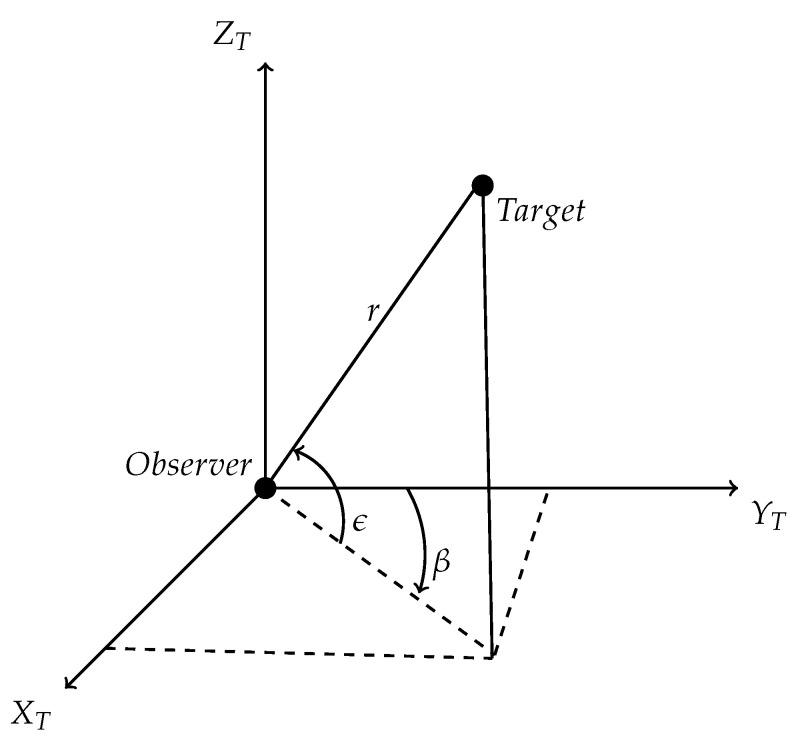
Target Observer in Cartesian Coordinate Frame.

**3D AoT problem:**Figure 3 represents the target observer dynamics in Cartesian coordinate.

The range vector r is defined as

$$\begin{array}{cc} r & ={\left[\begin{array}{ccc} x_{k}^{t}-x_{k}^{o} & y_{k}^{t}-y_{k}^{o}\; & z_{k}^{t}-z_{k}^{o} \end{array}\right]}^{\prime }\\ \; & ={\left[\begin{array}{ccc} x_{k} & y_{k} & z_{k} \end{array}\right]}^{\prime }. \end{array}$$

From Figure 3, r can be expressed in terms of bearing (β) and elevation (ϵ) as

$$r={\left[r\cos\epsilon \sin\beta \;\;\;r\cos\epsilon \cos\beta \;\;\;r\sin\epsilon \right]}^{\prime },$$

and the actual range is $r_{k}=\sqrt{x_{k}^{2}+y_{k}^{2}+z_{k}^{2}}$. Here, the nonlinear noise corrupted measurements are bearing (β) and elevation (ϵ) angles respectively, where β∈[0,2π] and $\epsilon \;\in [-\frac{\pi }{2},\frac{\pi }{2}]$. The measurement model involving the bearing and elevation angle is

(5) $$\begin{array}{c} z_{k}=h\left(X_{k}\right)+v_{k}, \end{array}$$

where,

$$\begin{array}{c} h\left(X_{k}\right)=\left[\begin{array}{c} \beta_{k}\\ \epsilon_{k} \end{array}\right]=\left[\begin{array}{c} \tan^{-1}\left(x_{k},y_{k}\right)\\ \tan^{-1}\left(\frac{z_{k}}{\sqrt{{x_{k}}^{2}+{y_{k}}^{2}}}\right) \end{array}\right]. \end{array}$$

Here, $v_{k}$ is to be modelled as the non Gaussian noise.

## 3. Correntropy Measure

Correntropy is directly related to the probability of how similar two random variables are in the joint space controlled by the kernel bandwidth. The kernel bandwidth controls the window in which the similarity has to be assessed, and hence provides a way to eliminate the detrimental effect of outliers [16]. If *X* and *Y* are random variables, correntropy is defined as

$$V_{\sigma }\left(X,Y\right)=E\left[k_{\sigma }\left(X,Y\right)\right]=\;\int \int \;k_{\sigma }\left(x,y\right)p_{XY}\left(x,y\right)\;dx\;dy,$$

where $k_{\sigma }$ denotes a positive definite kernel function, $p_{XY}\left(.\right)$ denotes the joint PDF of *X* and *Y* and *E* is the expectation operator. Since the joint density is not accessible and if only a finite number of data points *N* are available, a sample estimator can be defined as

$${\hat{V}}_{\sigma }\left(X,Y\right)=\frac{1}{N}\sum_{i=1}^{N}G_{\sigma }\left(x_{i}-y_{i}\right).$$

Here $G_{\sigma }\left(\cdot \right)$ is the Gaussian kernel, defined as

(6) $$G_{\sigma }\left(x_{i}-y_{i}\right)=\exp\left(-\frac{∥x_{i}-y_{i}{∥}^{2}}{2\sigma^{2}}\right),$$

which is bounded, positive and reaches its maximum only when X=Y, leading to the maximum correntropy criterion (MCC). By taking the Taylor series expansion of the Gaussian kernel, correntropy can also be expressed as a weighted sum of all even order moments of $\left(x_{i}-y_{i}\right)$, i.e.,

$$V_{\sigma }\left(X,Y\right)=\sum_{k=0}^{\infty }\frac{{\left(-1\right)}^{k}}{2^{k}\sigma^{2k}k!}E\left[{\left(X-Y\right)}^{2k}\right].$$

On the other hand, Cauchy kernel based non-linear state estimators can be developed using Cauchy kernel instead of Gaussian kernel function. It is defined as [34]

$$C_{\delta }\left(x_{i}-y_{i}\right)=\frac{1}{1+\frac{∥x_{i}-y_{i}{∥}^{2}}{\delta }}.$$

Here δ is a positive scalar, representing the Cauchy kernel bandwidth. Similar to the Gaussian kernel, it can be shown that the Cauchy kernel also incorporates the higher order moments, given as

Vδ(X,Y)=∑k=0∞(−1)kδkN+k−1kE(X−Y)2k.

A detailed derivation of the above equation is given in Appendix A.

## 4. Gaussian Kernel Based Maximum Correntropy Estimation Framework

Let us consider the process model described in Equations (1) and (5). To accommodate for the large outliers in measurements, the noise $v_{k}$ is considered non-Gaussian. Hence for MC based estimation framework [24], the Gaussian assumption of $v_{k}$ is relaxed.

In order to deal with the non Gaussian noises in the measurement update step, a statistical linearisation approach is employed. Consider that the nonlinear function h(·), operating on vector random variables $X_{k}$ is evaluated at N-points $\chi_{k},\;k=1,\cdots ,N$, with $z_{k}=h\left(\chi_{k}\right)+v_{k}$. Suppose that the weighted mean of $\chi_{k}$ is given by ${\hat{X}}_{k|k-1}=\sum_{k=1}^{N}W_{k}\chi_{k}$, with $\sum_{k=1}^{N}W_{k}=1$. Similarly, ${\hat{z}}_{k|k-1}=\sum_{k=1}^{N}W_{k}z_{k}$. Then the prior and cross covariance $P_{k|k-1}$ and $P_{Xz}$ are given as

$$\begin{array}{cc} P_{k|k-1} & =\sum_{k=1}^{N}W_{k}\left[\left(\chi_{k}-{\hat{X}}_{k|k-1}\right){\left(\chi_{k}-{\hat{X}}_{k|k-1}\right)}^{\prime }\right]\;and\\ P_{Xz} & =\sum_{k=1}^{N}W_{k}\left[\left(\chi_{k}-{\hat{X}}_{k|k-1}\right){\left(z_{k}-{\hat{z}}_{k|k-1}\right)}^{\prime }\right]. \end{array}$$

The nonlinear measurement function is represented in terms of measurement slope matrix ${\bar{H}}_{k}$, and a constant term ${\bar{c}}_{k}$ as $h\left(X_{k}\right)\approx {\bar{H}}_{k}X_{k}+{\bar{c}}_{k}$. Here ${\bar{H}}_{k}$ and ${\bar{c}}_{k}$ are computed by minimizing the weighted least squares,

$$\underset{{\bar{H}}_{k},{\bar{c}}_{k}}{\arg\;\min}\;W_{k}{∥{\bar{v}}_{k}∥}^{2},\;where\;{\bar{v}}_{k}=z_{k}-{\bar{H}}_{k}X_{k}-{\bar{c}}_{k}.$$

Then the solutions are ${\bar{H}}_{k}={\left(P_{k|k-1}^{-1}P_{Xz}\right)}^{\prime }\;and\;{\bar{c}}_{k}={\hat{z}}_{k|k-1}-{\bar{H}}_{k}{\hat{X}}_{k|k-1}$. As the mean of ${\bar{v}}_{k}$ is zero, that is $E\left[{\bar{v}}_{k}\right]={\hat{z}}_{k|k-1}-{\bar{H}}_{k}{\hat{X}}_{k|k-1}-{\bar{c}}_{k}=0$, the covariance matrix $R_{k}$ can be calculated as

(7) $$\begin{array}{cc} R_{k}= & \sum_{k=1}^{N}W_{k}\left[{\bar{v}}_{k}{\bar{v}}_{k}^{\prime }\right]\\ = & \sum_{k=1}^{N}W_{k}\left[\left(z_{k}-{\hat{z}}_{k|k-1}\right)-{\bar{H}}_{k}\left(X_{k}-{\hat{X}}_{k|k-1}\right)\right]{\left[\left(z_{k}-{\hat{z}}_{k|k-1}\right)-{\bar{H}}_{k}\left(X_{k}-{\hat{X}}_{k|k-1}\right)\right]}^{\prime }\\ = & P_{zz}-{\bar{H}}_{k}P_{Xz}-P_{Xz}^{\prime }{\bar{H}}_{k}^{\prime }+{\bar{H}}_{k}P_{k|k-1}{\bar{H}}_{k}^{\prime }\\ = & P_{zz}-{\bar{H}}_{k}P_{k|k-1}{\bar{H}}_{k}^{\prime }. \end{array}$$

Thus, the linearised measurement equation is given as

(8) $$z_{k}={\hat{z}}_{k|k-1}+{\bar{H}}_{k}\left(X_{k}-{\hat{X}}_{k|k-1}\right)+{\bar{v}}_{k}\;\mathrm{with}\;{\bar{v}}_{k}∼N\left(0,R_{k}\right).$$

Accordingly, a cost function is formulated with the help of weighted least squares (WLS) to handle Gaussian process noise. To handle non-Gaussian measurement noise, statistical linearisation approach was used to define WLS function which in turn is used in MCC. Hence the cost function can be defined as

$$J=℘{∥X_{k}-{\hat{X}}_{k|k-1}∥}_{P_{k|k-1}^{-1}}^{2}-\varrho \exp\left(-\frac{{ℵ}^{\prime }R^{-1}ℵ}{2\sigma^{2}}\right),$$

where $ℵ=z_{k}-{\hat{z}}_{k|k-1}-{\bar{H}}_{k}\left(X_{k}-{\hat{X}}_{k|k-1}\right)$, *℘* and ϱ are adjustable weights. In order to find the optimal estimate of $X_{k}$, the cost function has to be minimized i.e.,

$${\hat{X}}_{k}=\underset{X_{k}}{\arg\;\min}\;J,$$

and the solution can be obtained as $\frac{\partial J}{\partial X_{k}}=0$. This implies that

(9) $$\begin{array}{cc} \frac{\partial J}{\partial X_{k}} & =℘P_{k|k-1}^{-1}\left(X_{k}-{\hat{X}}_{k|k-1}\right)+\frac{\varrho }{2\sigma^{2}}G_{\sigma }\left({ℵ}_{R}\right){\bar{H}}_{k}^{\prime }R_{k}^{-1}ℵ\\ \; & =℘P_{k|k-1}^{-1}\left(X_{k}-{\hat{X}}_{k|k-1}\right)+\frac{\varrho L_{k}^{G}{\bar{H}}_{k}^{\prime }R_{k}^{-1}}{2\sigma^{2}}ℵ=0, \end{array}$$

where

(10) $$\begin{array}{c} L_{k}^{G}=G_{\sigma }\left({ℵ}_{R}\right)=\exp\left(-\frac{{ℵ}^{\prime }R^{-1}ℵ}{2\sigma^{2}}\right). \end{array}$$

In order to guarantee the convergence of the algorithm to a corresponding state estimator that follows a complete Gaussian assumption (when the kernel bandwidth σ becomes infinity), the values for weights in J are taken as ℘=1 and $\varrho =-2\sigma^{2}$. Then Equation (9) becomes

$$P_{k|k-1}^{-1}\left(X_{k}-{\hat{X}}_{k|k-1}\right)=L_{k}^{G}{\bar{H}}_{k}^{\prime }R_{k}^{-1}ℵ.$$

Rearranging, we get

(11) $$\left(P_{k|k-1}^{-1}+L_{k}^{G}{\bar{H}}_{k}^{\prime }R_{k}^{-1}{\bar{H}}_{k}\right)X_{k}=L_{k}^{G}{\bar{H}}_{k}^{\prime }R_{k}^{-1}\left(z_{k}-{\hat{z}}_{k|k-1}\right)+\left(L_{k}^{G}{\bar{H}}_{k}^{\prime }R_{k}^{-1}{\bar{H}}_{k}+P_{k|k-1}^{-1}\right){\hat{X}}_{k|k-1}.$$

Since $L_{k}^{G}$ is related to $X_{k}$, Equation (11) represents a fixed point equation that can be solved using the fixed point iteration algorithm considering $X_{k}$ equal to ${\hat{X}}_{k|k-1}$ in Equation (10). But as mentioned in [19,22,24], for a satisfactory estimation performance, a single iteration is sufficient. Hence, adopting the same approach leads to the modification of Equation (11) as

$${\hat{X}}_{k|k}={\hat{X}}_{k|k-1}+K_{k}^{G}\left(z_{k}-{\hat{z}}_{k|k-1}\right),$$

where

$$\begin{array}{cc} K_{k}^{G} & ={\left(P_{k|k-1}^{-1}+L_{k}^{G}{\bar{H}}_{k}^{\prime }R_{k}^{-1}{\bar{H}}_{k}\right)}^{-1}L_{k}^{G}{\bar{H}}_{k}^{\prime }R_{k}^{-1}\;\mathrm{and}\\ L_{k}^{G} & =\exp\left(-\frac{{\left(z_{k}-{\hat{z}}_{k|k-1}\right)}^{\prime }R_{k}^{-1}\left(z_{k}-{\hat{z}}_{k|k-1}\right)}{2\sigma^{2}}\right). \end{array}$$

A more appropriate form for $K_{k}^{G}$, in terms of reduced computational complexity, can be derived using the matrix inversion lemma (detailed derivation is given in Appendix B) as

(12) $$K_{k}^{G}=P_{k|k-1}L_{k}^{G}{{\bar{H}}_{k}}^{\prime }{\left(R_{k}+{\bar{H}}_{k}P_{k|k-1}L_{k}^{G}{\bar{H}}_{k}^{\prime }\right)}^{-1}.$$

Now, the corresponding posterior error covariance matrix is given as

$$P_{k|k}=\left(I-K_{k}^{G}{\bar{H}}_{k}\right)P_{k|k-1}{\left(I-K_{k}^{G}{\bar{H}}_{k}\right)}^{\prime }+K_{k}^{G}R_{k}K_{k}^{G^{\prime }}.$$

## 5. Cauchy Kernel Based Maximum Correntropy Estimation Framework

In this section, we derive a maximum correntropy estimation framework using Cauchy kernel for potential improvement in estimation accuracy, in the presence of large multi dimensional non Gaussian noise. Hence the cost function becomes

$$J_{C}={℘}^{C}{∥X_{k}-{\hat{X}}_{k|k-1}∥}_{P_{k|k-1}^{-1}}^{2}-\varrho^{C}C_{\delta }\left({ℵ}_{R}\right)$$

where ${℘}^{C}$ and $\varrho^{C}$ are adjustable weights, and

$$C_{\delta }\left({ℵ}_{R}\right)=\frac{1}{1+\frac{{ℵ}^{\prime }R_{k}^{-1}ℵ}{\delta }},$$

with *ℵ* being the same as that mentioned in Section 4. To obtain the optimal estimate of $X_{k}$, we equate $\frac{\partial J_{C}}{\partial X_{k}}=0$, giving ${℘}^{C}P_{k|k-1}^{-1}\left(X_{k}-{\hat{X}}_{k|k-1}\right)-\frac{\varrho^{C}L_{k}^{C}}{\delta }{\bar{H}}^{\prime }R_{k}^{-1}ℵ=0$, where

$$L_{k}^{C}=C_{\delta }^{2}\left({ℵ}_{R}\right)=C_{\delta }^{2}\left({∥z_{k}-{\bar{H}}_{k}X_{k}-{\hat{z}}_{k|k-1}+{\bar{H}}_{k}{\hat{X}}_{k|k-1}∥}_{R_{k}^{-1}}\right).$$

We set ${℘}^{C}=1$ and $\varrho^{C}=\delta$ so as to guarantee the convergence of the estimator when kernel bandwidth δ tends to *∞*. Rearranging,

(13) $$\left(P_{k|k-1}^{-1}+L_{k}^{C}{\bar{H}}_{k}^{\prime }R_{k}^{-1}{\bar{H}}_{k}\right)X_{k}=L_{k}^{C}{\bar{H}}_{k}^{\prime }R_{k}^{-1}\left(z_{k}-{\hat{z}}_{k|k-1}\right)+\left(L_{k}^{C}{\bar{H}}_{k}^{\prime }R_{k}^{-1}{\bar{H}}_{k}+P_{k|k-1}^{-1}\right){\hat{X}}_{k|k-1}.$$

Here also, $L_{k}^{C}$ is related to $X_{k}$ and hence Equation (13) is a fixed point equation that is to be solved using fixed point iteration algorithm, considering $X_{k}$ equal to ${\hat{X}}_{k|k-1}$. Using the same justification that was adopted in Gaussian kernel case that only a single iteration is required, the expression for posterior mean is obtained as

$${\hat{X}}_{k|k}={\hat{X}}_{k|k-1}+K_{k}^{C}\left(z_{k}-{\hat{z}}_{k|k-1}\right),$$

where

$$K_{k}^{C}={\left(P_{k|k-1}^{-1}+L_{k}^{C}{\bar{H}}_{k}^{\prime }R_{k}^{-1}{\bar{H}}_{k}\right)}^{-1}L_{k}^{C}{\bar{H}}_{k}^{\prime }R_{k}^{-1}\;\mathrm{and}\;L_{k}^{C}=C_{\delta }^{2}\left({∥z_{k}-{\hat{z}}_{k|k-1}∥}_{R_{k}^{-1}}\right).$$

As per the proof given in Appendix B, Kalman gain can be modified as

(14) $$K_{k}^{C}=P_{k|k-1}L_{k}^{C}{\bar{H}}_{k}^{\prime }{\left(R_{k}+{\bar{H}}_{k}P_{k|k-1}L_{k}^{C}{\bar{H}}_{k}^{\prime }\right)}^{-1}.$$

Then the posterior error covariance matrix shall be calculated as

(15) $$P_{k|k}=\left(I-K_{k}^{C}{\bar{H}}_{k}\right)P_{k|k-1}{\left(I-K_{k}^{C}{\bar{H}}_{k}\right)}^{\prime }+K_{k}^{C}R_{k}K_{k}^{C^{\prime }}.$$

**Theorem** **1.** *As the kernel bandwidth δ→∞, the Cauchy kernel based MC estimator reduces to the standard nonlinear state estimation algorithm.*

**Proof.**  As the time update is the same for the developed algorithms with respect to the standard nonlinear state estimators, the prior mean and covariance is unchanged. Hence the focus shall be on the posterior mean and covariance. This implies that the Kalman gain equation has to be revisited. When δ→∞,

(16) $$\lim_{\delta \to \infty }L_{k}^{C}=\lim_{\delta \to \infty }C_{\delta }^{2}\left({∥z_{k}-{\hat{z}}_{k|k-1}∥}_{R_{k}^{-1}}\right)=\lim_{\delta \to \infty }\frac{1}{{\left(1+\frac{{ℵ}^{\prime }R_{k}^{-1}ℵ}{\delta }\right)}^{2}}=1.$$

Substituting the Equations (7) and (16) and ${\bar{H}}_{k}$ in $K_{k}^{C}$, we have

$$K_{k}^{C}=P_{k|k-1}\left(P_{k|k-1}^{-1}P_{Xz}\right){\left(P_{zz}-{\bar{H}}_{k}P_{k|k-1}{\bar{H}}_{k}^{\prime }+{\bar{H}}_{k}P_{k|k-1}{\bar{H}}_{k}^{\prime }\right)}^{-1}=P_{Xz}{P_{zz}}^{-1}.$$

Since the expression of $K_{k}^{C}$ is similar to the Kalman gain of standard nonlinear state estimator, posterior mean is also the same.

Now, for the posterior covariance $P_{k|k}$, consider Equation (15),

(17) $$P_{k|k}=P_{k|k-1}-P_{k|k-1}{\bar{H}}_{k}^{\prime }K_{k}^{C^{\prime }}-K_{k}^{C}{\bar{H}}_{k}P_{k|k-1}+K_{k}^{C}\left(R_{k}+{\bar{H}}_{k}P_{k|k-1}L_{k}^{C}{\bar{H}}_{k}^{\prime }\right)K_{k}^{C^{\prime }}.$$

Post multiplying Equation (14) by $\left(R_{k}+{\bar{H}}_{k}P_{k|k-1}L_{k}^{C}{\bar{H}}_{k}^{\prime }\right)$ on both sides give

(18) $$K_{k}^{C}\left(R_{k}+{\bar{H}}_{k}P_{k|k-1}L_{k}^{C}{\bar{H}}_{k}^{\prime }\right)=P_{k|k-1}L_{k}^{C}{\bar{H}}_{k}^{\prime }.$$

Using Equations (16) and (18)

$$P_{k|k}=P_{k|k-1}-P_{k|k-1}{\bar{H}}_{k}^{\prime }K_{k}^{C^{\prime }}-K_{k}^{C}{\bar{H}}_{k}P_{k|k-1}+P_{k|k-1}{\bar{H}}_{k}^{\prime }K_{k}^{C^{\prime }}=P_{k|k-1}-K_{k}^{C}{\bar{H}}_{k}P_{k|k-1}.$$

Substituting ${\bar{H}}_{k}$, we get $P_{k|k}=P_{k|k-1}-K_{k}^{C}P_{Xz}^{\prime }$. For the given condition, $K_{k}^{C}=P_{Xz}{P_{zz}}^{-1}$, then $P_{Xz}^{\prime }={P_{zz}}^{\prime }K_{k}^{C^{\prime }}$. Thus $P_{k|k}$ will become $P_{k|k}=P_{k|k-1}-K_{k}^{C}{P_{zz}}^{\prime }K_{k}^{C^{\prime }}$, which matches with the posterior error covariance of standard nonlinear estimator.    □

**Remark** **1.** *For systems with non-Gaussian noise with large probability of abnormal noise, small value of δ is likely to provide more robustness. If the occurrence of abnormal noise is less, large value of δ could be considered.*

**Remark** **2.** *Cauchy kernel based nonlinear estimator with different δ performs with more estimation accuracy than Gaussian kernel based nonlinear estimator with different σ. Hence it is easier to select a value for δ that can provide accurate and robust estimates in the presence of abnormal noise.*

## 6. Nonlinear State Estimators

This section deals with the nonlinear state estimators UKF and NSKF with a generalized algorithm for kernel based MC estimator.

### 6.1. Unscented Kalman Filter (UKF)

In the Bayesian framework, when the functions are nonlinear, the integrals encountered are intractable in nature and has to be evaluated using suitable numerical approximation methods. The UKF, through its unscented transformation, provides a way to numerically evaluate these integrals. Assuming that the integral to be approximated is

$$I\left(X\right)=\int h\left(X\right)p_{X}\left(X\right)dX,$$

and $X∼N(\hat{X},P)$, the unscented transformation defines a set of sigma points (${\hat{X}}_{i}$) and weights ($W_{i}$) such that [35]

$I\left(X\right)≊\sum_{i=1}^{N}W_{i}h\left({\hat{X}}_{i}\right)$, where *n* is the dimension of the state space and **N** = 2*n* + 1.

The sigma points and weights are defined as

(19) $$\begin{array}{cc} {\hat{X}}_{1} & =\hat{X},\;W_{1}=\frac{\kappa }{n+\kappa },\\ {\hat{X}}_{i} & =\hat{X}+{\left(\sqrt{(n+\kappa )P}\right)}_{i}\;W_{i}=\frac{1}{2(n+\kappa )},\;i=1,\cdots ,n\\ {\hat{X}}_{i} & =\hat{X}-{\left(\sqrt{(n+\kappa )P}\right)}_{i}\;W_{i}=\frac{1}{2(n+\kappa )},\;i=1,\cdots ,n, \end{array}$$

with κ being the tuning parameter and $\hat{X}$ is the mean.

### 6.2. New Sigma Point Kalman Filter (NSKF)

From Equation (19), it can observed that in the unscented transformation, the maximum weight is assigned to the mean value. All the other sigma points are assigned equal weights, i.e., same probability of occurrence. In NSKF, a new approach was considered such that the sigma points closer to the mean will have more probability of occurrence. To realize this, a new method was formulated for defining the sigma points and weights, stated as [8]

(20) $$\begin{array}{cc} \; & {\hat{X}}_{1}=\hat{X},\;W_{1}=1-\frac{\sum_{i=1}^{n}\alpha_{i}}{2(\sum_{i=1}^{n}\alpha_{i}+b)}\\ \; & {\hat{X}}_{i+1}=\hat{X}+\sqrt{\frac{\sum_{i=1}^{n}\alpha_{i}+b}{m\alpha_{i}}}S_{i},\;W_{i+1}=\frac{m\alpha_{i}}{4(\sum_{i=1}^{n}\alpha_{i}+b)},\;i=1,\cdots ,n\\ \; & {\hat{X}}_{i+1}=\hat{X}-\sqrt{\frac{\sum_{i=1}^{n}\alpha_{i}+b}{m\alpha_{i-n}}}S_{i-n},\;W_{i+1}=\frac{m\alpha_{i-n}}{4(\sum_{i=1}^{n}\alpha_{i}+b)},\;i=n+1,\cdots ,2n\\ \; & {\hat{X}}_{i+1}=\hat{X}+\sqrt{\frac{\sum_{i=1}^{n}\alpha_{i}+b}{\left(1-m\right)\alpha_{i-2n}}}S_{i-2n},\;W_{i+1}=\frac{\left(1-m\right)\alpha_{i-2n}}{4(\sum_{i=1}^{n}\alpha_{i}+b)},\;i=2n+1,\cdots ,3n\\ \; & {\hat{X}}_{i+1}=\hat{X}-\sqrt{\frac{\sum_{i=1}^{n}\alpha_{i}+b}{\left(1-m\right)\alpha_{i-3n}}}S_{i-3n},\;W_{i+1}=\frac{\left(1-m\right)\alpha_{i-3n}}{4(\sum_{i=1}^{n}\alpha_{i}+b)},\;i=3n+1,\cdots ,4n. \end{array}$$

Now the total number of sigma points N=4n+1, $P_{i}\;\mathrm{and}\;S_{i}$ denote the *i*th column of PandS respectively, and $SS^{\prime }=P$. The scalar variables are defined as $b>\{\frac{1}{4}\max\left(m\alpha_{i}\right)-\frac{1}{2}\sum_{i=1}^{n}\alpha_{i}\}$, m∈(0.5,1) and $\alpha_{i}=\frac{|<\hat{X},P_{i}>|}{\|\hat{X}{\|}_{2}{\left\|P_{i}\right\|}_{2}}$.

The Algorithm 1 for the developed estimators, both Gaussian kernel and Cauchy kernel based is given below. In this algorithm, $K_{k}$ and $L_{k}$ can be defined as per the chosen kernel function. $R_{k}$ is the noise covariance matrix which is assumed to be known in case there are no measurement outliers.
**Algorithm 1:** For MC-UKF-CK and MC-NSKF-CKInitialise${\hat{X}}_{k-1|k-1}$and$P_{k-1|k-1}$${\hat{X}}_{k|k-1}=F_{k-1}{\hat{X}}_{k-1|k-1}-X_{k}^{o}+F_{k-1}X_{k-1}^{o}$$P_{k|k-1}=F_{k-1}P_{k-1|k-1}F_{k-1}^{\prime }+Q$Calculate${\hat{X}}_{i}$ and $W_{i}$using (19) or (20), i=1,⋯,N$Z_{i,k|k-1}=h\left({\hat{X}}_{i}\right)$${\hat{z}}_{k}=\sum_{i=1}^{N}W_{i}Z_{i,k|k-1}$$P_{zz}=\sum_{i=1}^{N}W_{i}\left[Z_{i,k|k-1}-{\hat{z}}_{k}\right]{\left[Z_{i,k|k-1}-{\hat{z}}_{k}\right]}^{\prime }+R_{k}$$P_{Xz}=\sum_{i=1}^{N}W_{i}\left[{\hat{X}}_{i,k|k-1}-{\hat{X}}_{k|k-1}\right]{\left[Z_{i,k|k-1}-{\hat{z}}_{k}\right]}^{\prime }$${\bar{H}}_{k}={\left(P_{k|k-1}^{-1}P_{Xz}\right)}^{\prime }$$R_{k}=P_{zz}-{\bar{H}}_{k}P_{k|k-1}{\bar{H}}_{k}^{\prime }$$K_{k}=P_{k|k-1}L_{k}{\bar{H}}_{k}^{\prime }{\left(R_{k}+{\bar{H}}_{k}P_{k|k-1}L_{k}{\bar{H}}_{k}^{\prime }\right)}^{-1}.$**Posterior mean**: ${\hat{X}}_{k|k}={\hat{X}}_{k|k-1}+K_{k}\left(z_{k}-{\hat{z}}_{k}\right)$.**Posterior covariance**: $P_{k|k}=\left(I-K_{k}{\bar{H}}_{k}\right)P_{k|k-1}{\left(I-K_{k}{\bar{H}}_{k}\right)}^{\prime }+K_{k}R_{k}K_{k}^{\prime }$.

## 7. Modelling of Non Gaussian Noise in Angular Measurements

As mentioned in [36], a suitable way of modelling glint noise is to assume a Gaussian mixture. It is observed that the glint is more like Gaussian around the mean but has a non-Gaussian nature towards the tail region. The tail region represents the outliers, termed as glint spikes [32]. But shot noise, on the other hand, is modelled as an impulse with fixed amplitude at specific time steps. The mixture density of glint noise is modelled as $f\left(x\right)=\left(1-\mu \right)f_{g1}\left(x\right)+\mu f_{g2}\left(x\right)$, where μ is the glint probability and $f_{g1}\left(x\right)∼N\left(0,\sigma_{1}^{2}\right)$, $f_{g2}\left(x\right)∼N\left(0,\sigma_{2}^{2}\right)$ with $\sigma_{1}\neq \sigma_{2}$. The non Gaussian noises for angular measurements have been modelled by taking appropriate values for μ, $\sigma_{1}$ and $\sigma_{2}$.

## 8. Simulation Results

The scenario for angles-only tracking problem in 2D as well as 3D Cartesian coordinate frame is considered in this section. The parameters required for generating the target-observer dynamics, and simulation results are discussed. For simulations, moderately nonlinear tracking scenario for 2D as well as for 3D is considered. The simulation is carried for 1000 Monte Carlo runs with sampling time interval denoted as *T*. The entire tracking scenario is implemented and simulated in MATLAB software.

### 8.1. 2D Scenario and Filter Initialisation

**Figure 4:**
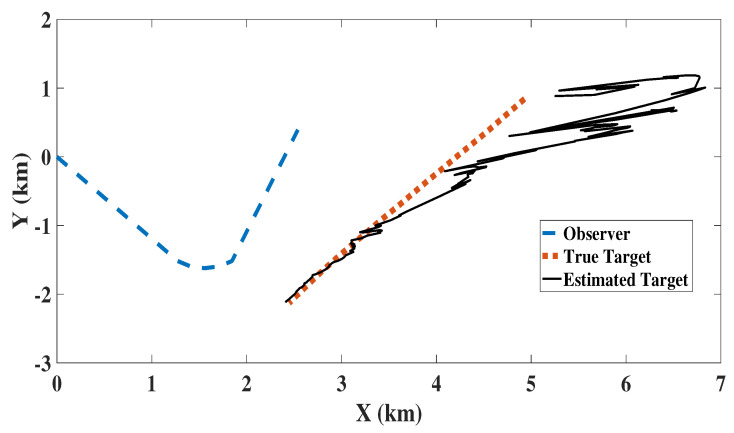
Target truth and estimated path obtained from MC-NSKF-CK.

Figure 4 shows the tracking performance of MC-NSKF-CK, where the estimated target path is plotted along with the truth target path, and the observer path for a single Monte Carlo run. It should be noted that for each run, observer path remains the same where as the target path varies due to the process noise. Further, the filter initialisation is also changing because of the randomness introduced in each run, as mentioned in Equation (21).

The filter is initialised as given in [10]. It is to be noted that for filter initialisation, we need an initial guess for speed, initial course and range of the target. Considering the problem at hand, these estimates have to be obtained from the initial angle measurement received. From these initial guess for parameters, the initial estimate for the states are obtained which are the positions and velocities. Accordingly, the initial range, target course and speed values are considered and mentioned in the Table 1. They are defined as $\bar{s}=N\left(s,\sigma_{s}^{2}\right)$, $c_{r}=N\left({\bar{c}}_{r},\sigma_{c}^{2}\right)$ and $\bar{r}=N\left(r,\sigma_{r}^{2}\right)$ where ${\bar{c}}_{r}$ can be defined as ${\bar{c}}_{r}=z_{0}+\pi$ with $z_{0}$ as the first bearing measurement. Finally the initial state vector ${\hat{X}}_{0|0}$ and the initial covariance $P_{0|0}$ is calculated as

(21) $$\begin{array}{cccc} {\hat{X}}_{0|0} & =\left[\begin{array}{c} r\sin(z_{0})\\ r\cos(z_{0})\\ s\sin\left({\bar{c}}_{r}\right)-{\dot{x}}_{0}^{o}\\ s\cos\left({\bar{c}}_{r}\right)-{\dot{y}}_{0}^{o} \end{array}\right] & P_{0|0} & =\left[\begin{array}{cccc} P_{\mathrm{xx}} & P_{\mathrm{xy}} & 0 & 0\\ P_{\mathrm{yx}} & P_{\mathrm{yy}} & 0 & 0\\ 0 & 0 & P_{\dot{x}\dot{x}} & P_{\dot{x}\dot{y}}\\ 0 & 0 & P_{\dot{y}\dot{x}} & P_{\dot{y}\dot{y}} \end{array}\right] \end{array}$$

where

$$\begin{array}{cccc} P_{\mathrm{xx}} & =r^{2}\sigma_{\beta }^{2}\cos^{2}\left(z_{0}\right)+\sigma_{r}^{2}\sin^{2}\left(z_{0}\right) & P_{\mathrm{yy}} & =r^{2}\sigma_{\beta }^{2}\sin^{2}\left(z_{0}\right)+\sigma_{r}^{2}\cos^{2}\left(z_{0}\right)\\ P_{\mathrm{xy}} & =P_{\mathrm{yx}}=\left(\sigma_{r}^{2}-r^{2}\sigma_{\beta }^{2}\right)\sin\left(z_{0}\right)\cos\left(z_{0}\right) & P_{\dot{x}\dot{x}} & =s^{2}\sigma_{c}^{2}\cos^{2}\left({\bar{c}}_{r}\right)+\sigma_{s}^{2}\sin^{2}\left({\bar{c}}_{r}\right)\\ P_{\dot{y}\dot{y}} & =s^{2}\sigma_{c}^{2}\sin^{2}\left({\bar{c}}_{r}\right)+\sigma_{s}^{2}\cos^{2}\left({\bar{c}}_{r}\right) & P_{\dot{x}\dot{y}} & =P_{\dot{y}\dot{x}}=\left(\sigma_{s}^{2}-s^{2}\sigma_{c}^{2}\right)\sin\left({\bar{c}}_{r}\right)\cos\left({\bar{c}}_{r}\right). \end{array}$$

### 8.2. 3D Scenario and Filter Initialisation

**Figure 5:**
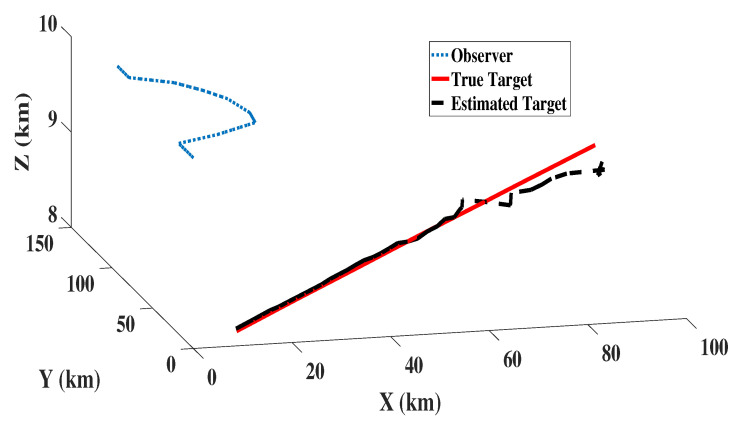
Target truth and estimated path obtained from MC-NSKF-CK.

Figure 5 shows the estimated target path obtained from MC-NSKF-CK and truth target path with observer trajectory. The initial parameter values required for generating the 3D scenario is given in the Table 2. Assuming that there are no outliers in the measurement, $R_{k}$ is defined as $R_{k}=diag\left(\sigma_{\beta },\sigma_{\epsilon }\right)$. The bearing angle β is calculated with reference to the true North.

For each Monte Carlo run, according to the new measurement received, initial range *r* and speed of the target *s* is assumed as mentioned in Table 2. According to these values, the relative state is initialised using the range estimate $\bar{r}∼N\left(r,{\sigma_{r}}^{2}\right)$, initial bearing and elevation estimate ${\hat{\beta }}_{1}$ and ${\hat{\epsilon }}_{1}$ with headings ${\bar{\alpha }}_{1}=\beta_{1}+\pi$ rad/s and ${\bar{\gamma }}_{1}=0$ rad/s, and the initial speed estimate $\bar{s}∼N\left(s,{\sigma_{s}}^{2}\right)$ with *s* as 0.258 km/s. The $\sigma_{{\bar{\alpha }}_{1}}=\pi /\sqrt{12}$ and $\sigma_{{\bar{\gamma }}_{1}}=\pi /60$ respectively [37].

The initial relative state vector ${\hat{X}}_{0|0}$ is given as [38]

$${\hat{X}}_{0|0}=\left[\begin{array}{c} \bar{r}\;\zeta_{1,0}\left({\hat{\epsilon }}_{1},{\sigma_{\epsilon }}^{2}\right)\;\zeta_{0,1}\left({\hat{\beta }}_{1},{\sigma_{\beta }}^{2}\right)\\ \bar{r}\;\zeta_{1,0}\left({\hat{\epsilon }}_{1},{\sigma_{\epsilon }}^{2}\right)\;\zeta_{1,0}\left({\hat{\beta }}_{1},{\sigma_{\beta }}^{2}\right)\\ \bar{r}\;\zeta_{0,1}\left({\hat{\epsilon }}_{1},{\sigma_{\epsilon }}^{2}\right)\\ \bar{s}\;\zeta_{1,0}\left({\bar{\gamma }}_{1},{\sigma_{\gamma }}^{2}\right)\;\zeta_{0,1}\left({\bar{\alpha }}_{1},{\sigma_{\alpha }}^{2}\right)-{\dot{x}}_{1}^{o}\\ \bar{s}\;\zeta_{1,0}\left({\bar{\gamma }}_{1},{\sigma_{\gamma }}^{2}\right)\;\zeta_{1,0}\left({\bar{\alpha }}_{1},{\sigma_{\alpha }}^{2}\right)-{\dot{y}}_{1}^{o}\\ \bar{s}\;\zeta_{0,1}\left({\bar{\gamma }}_{1},{\sigma_{\gamma }}^{2}\right)-{\dot{z}}_{1}^{o} \end{array}\right],$$

where $\zeta_{1,0}\left(\mu ,\sigma^{2}\right)=\cos\mu \;\exp\left(-\sigma^{2}/2\right)\;\mathrm{and}\;\zeta_{0,1}\left(\mu ,\sigma^{2}\right)=\sin\mu \;\exp\left(-\sigma^{2}/2\right)$.

The initial covariance matrix $P_{0|0}$, whose entries are considered as mentioned in [38], is defined as

$$P_{0|0}=\left[\begin{array}{cccccc} P_{\mathrm{xx}} & P_{\mathrm{xy}} & P_{\mathrm{xz}} & 0 & 0 & 0\\ P_{\mathrm{xy}} & P_{\mathrm{yy}} & P_{\mathrm{yz}} & 0 & 0 & 0\\ P_{\mathrm{xz}} & P_{\mathrm{yz}} & P_{\mathrm{zz}} & 0 & 0 & 0\\ 0 & 0 & 0 & P_{\dot{x}\dot{x}} & P_{\dot{x}\dot{y}} & P_{\dot{x}\dot{z}}\\ 0 & 0 & 0 & P_{\dot{x}\dot{y}} & P_{\dot{y}\dot{y}} & P_{\dot{y}\dot{z}}\\ 0 & 0 & 0 & P_{\dot{x}\dot{z}} & P_{\dot{y}\dot{z}} & P_{\dot{z}\dot{z}} \end{array}\right].$$

### 8.3. Performance Metrics

Performance analysis of the estimators formulated is evaluated by considering the below mentioned error statistics.

1. **RMSE:** Root-mean-square error in resultant target position is computed as follows

$$\begin{array}{cc} {\mathrm{RMSE}}_{k} & ={\sqrt{\frac{1}{M}\sum_{j=1}^{M}\left[{\left(x_{j,k}^{t}-{\hat{x}}_{j,k}^{t}\right)}^{2}+{\left(y_{j,k}^{t}-{\hat{y}}_{j,k}^{t}\right)}^{2}\right]}}_{n=2}\\ {\mathrm{RMSE}}_{k} & ={\sqrt{\frac{1}{M}\sum_{j=1}^{M}[{\left(x_{j,k}^{t}-{\hat{x}}_{j,k}^{t}\right)}^{2}+{\left(y_{j,k}^{t}-{\hat{y}}_{j,k}^{t}\right)}^{2}+{\left(z_{j,k}^{t}-{\hat{z}}_{j,k}^{t}\right)}^{2}]}}_{n=3} \end{array}$$

where *k* denotes the time steps and *M* the total number of Monte Carlo runs.

2. **Track Divergence:** In order to identify if a track is divergent or not, a certain threshold value ($T_{b}$) is set according to the position error computed at the final time instant of observation ($k_{max}$) as

$$\begin{array}{cc} {\mathrm{pos}}_{\mathrm{err}} & ={\sqrt{{\left(x_{j,k_{max}}^{t}-{\hat{x}}_{j,k_{max}}^{t}\right)}^{2}+{\left(y_{j,k_{max}}^{t}-{\hat{y}}_{j,k_{max}}^{t}\right)}^{2}}}_{n=2}\\ {\mathrm{pos}}_{\mathrm{err}} & ={\sqrt{{\left(x_{j,k_{max}}^{t}-{\hat{x}}_{j,k_{max}}^{t}\right)}^{2}+{\left(y_{j,k_{max}}^{t}-{\hat{y}}_{j,k_{max}}^{t}\right)}^{2}+{\left(z_{j,k_{max}}^{t}-{\hat{z}}_{j,k_{max}}^{t}\right)}^{2}}}_{n=3}\\ \; & \;for\;j=1,2,\cdots ,M. \end{array}$$

So, if the difference between estimated and truth target position is more than the threshold value ($T_{b}$), then we can say that the estimated path is moving away from the truth path. Thus, the track is considered to be divergent, and the number of such tracks are counted over *M* Monte Carlo runs.

### 8.4. Performance Analysis

The performance analysis of the developed filters is evaluated in the presence of glint plus shot noise in angle measurements. The accuracy of the estimators are evaluated by computing root mean square error (RMSE) in position at the end of the simulation period by imposing a track loss condition of 1 km.

(22) $$v_{k}=\left\{\begin{array}{cc} 0.2N\left(0,{\sigma_{\theta_{1}}}^{2}\right)+0.8N\left(0,{\sigma_{\theta_{2}}}^{2}\right)+10^{\circ }, & \mathrm{when}\;k=1200\;\mathrm{and}\;900\;s\\ 0.2N\left(0,{\sigma_{\theta_{1}}}^{2}\right)+0.8N\left(0,{\sigma_{\theta_{2}}}^{2}\right), & \mathrm{otherwise}. \end{array}\right.$$

**Figure 6:**
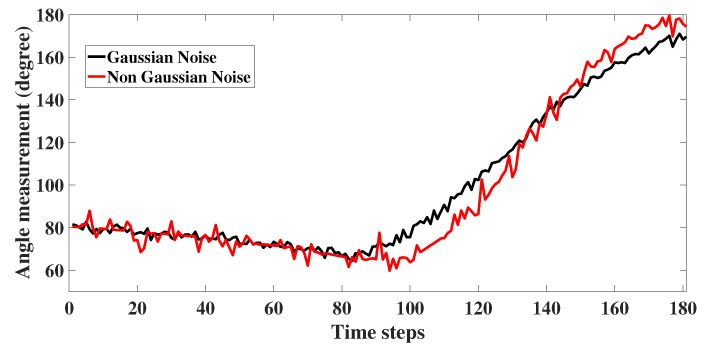
2D: Angle measurement with glint plus shot noise.

**Figure 7:**
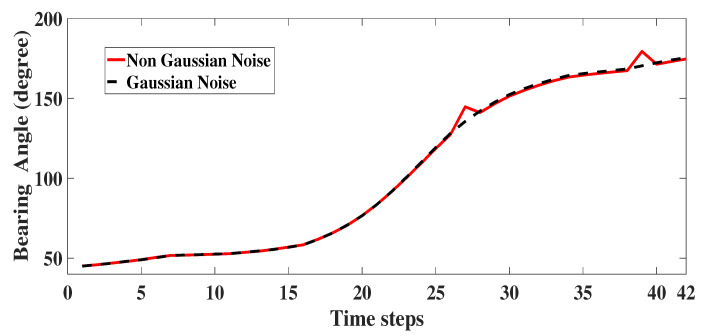
3D: Bearing angle measurement with glint plus shot noise.

**Figure 8:**
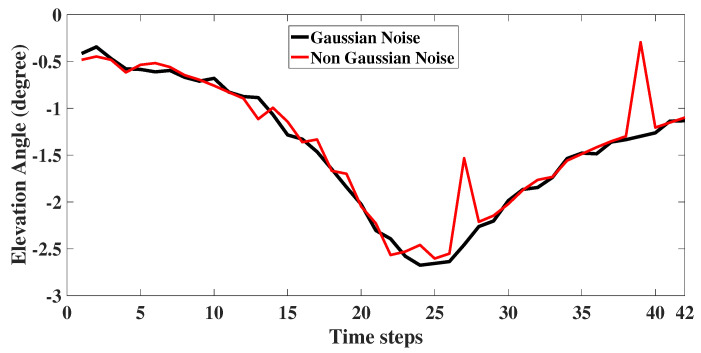
3D: Elevation angle measurement with glint plus shot noise.

The measurement noise $v_{k}$ for both the scenarios are given in Equations (22) and (23), respectively. Here, $\sigma_{\theta_{1}}=0.5^{\circ }$, $\sigma_{\theta_{2}}=5^{\circ }$, ($\sigma_{\beta_{1}},\sigma_{\epsilon_{1}}$) as 0.0001 rad and ($\sigma_{\beta_{2}},\sigma_{\epsilon_{2}}$) as 0.01 rad. The noise corrupted angle measurement for 2D is plotted as Figure 6. For 3D, the noise corrupted bearing and elevation angle are as shown in Figure 7 and Figure 8 respectively. For illustration, in the figures, we have also plotted the angle measurements with Gaussian noise.

(23) $$v_{k}=\left\{\begin{array}{c} 0.8N\left(0,diag\left(\left[{\sigma_{\beta_{1}}}^{2}\;{\sigma_{\epsilon_{1}}}^{2}\right]\right)\right)+0.2N\left(0,diag\left(\left[{\sigma_{\beta_{2}}}^{2}\;{\sigma_{\epsilon_{2}}}^{2}\right]\right)\right)+{\left[10^{\circ }\;\;1^{\circ }\right]}^{T},\\ \;\mathrm{when}\;k=270\;\mathrm{and}\;390\;s\\ 0.8N\left(0,diag\left(\left[{\sigma_{\beta_{1}}}^{2}\;{\sigma_{\epsilon_{1}}}^{2}\right]\right)\right)+0.2N\left(0,diag\left(\left[{\sigma_{\beta_{2}}}^{2}\;{\sigma_{\epsilon_{2}}}^{2}\right]\right)\right),\;\mathrm{otherwise}. \end{array}\right.$$

**Figure 9:**
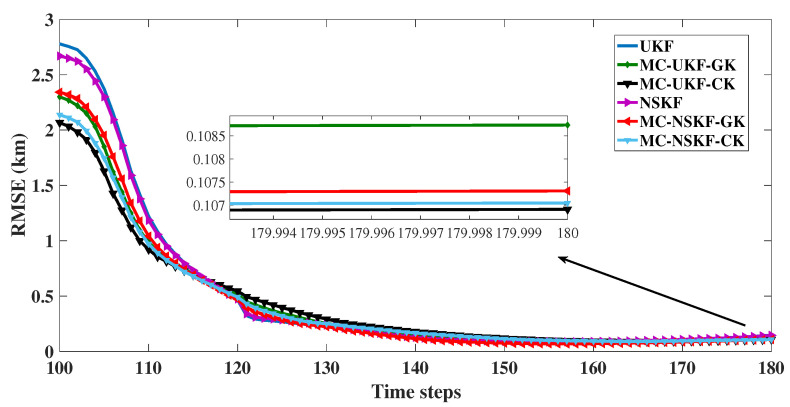
2D: RMSE in position.

**Figure 10:**
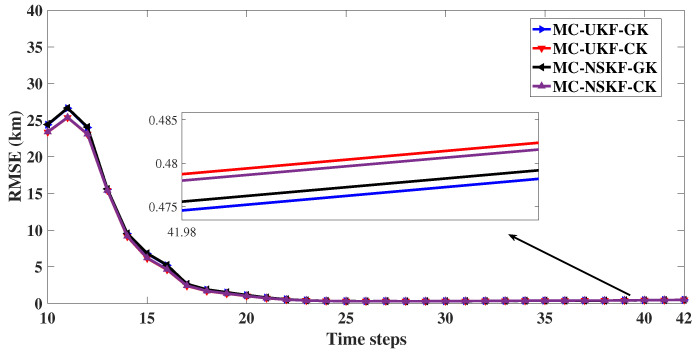
3D: RMSE in position.

With track loss condition of less than 1 km, the observed RMSE in position at the last time instant and percentage track loss for 2D as well as 3D is given in the Table 3 and Table 4 respectively. Also, RMSE in resultant position (after excluding the diverged tracks) is evaluated and plotted in Figure 9 and Figure 10. From these figures it can be inferred that in the presence of non Gaussian noise the estimation accuracy of UKF deteriorates, whereas filters based on MC framework performed with superior estimation accuracy. From the tabulation results it is evident that the Cauchy kernel based MC-UKF and MC-NSKF gives 108.9 m, 108.8 m RMSE and 1.1 and 0.5% track loss which is much less than that of the conventional UKF and NSKF which gives 152.8 m and 151.1 m RMSE with 4.4 and 2.8% track loss in 2D scenario. Similar observations can be made with respect to Gaussian kernel based MC-UKF and MC-NSKF giving much better accuracy but slightly less than Cauchy kernel MC framework. However, in 3D scenario, the MC based filters gave even superior estimation efficiency than that of the 2D scenario. UKF and NSKF in 3D with non Gaussian noise resulted in 100% track loss. Hence it can be inferred that for the given problem set up and noise statistics, UKF and NSKF failed to give estimates that met the track loss condition set, where as the Gaussian and Cauchy kernel based maximum correntropy filters gave more robust and accurate estimates. This can be inferred from the simulation results where the developed filters incurred only 13 to 14% track loss, with a final error in range of around 500 m. All these simulations are carried out by assuming the bandwidth (σ,δ) for 2D as (9,70) and for 3D as (11,75) such that the estimators can achieve maximum estimation accuracy. Also, the tuning parameter value of NSKF, m=0.6 is assumed for simulation.

## 9. Conclusions

Since, measurements obtained in target tracking scenarios are corrupted with non Gaussian noise, this paper presents a maximum correntropy framework for 2D as well as 3D angles-only target tracking problem. The reformulation of UKF and NSKF in terms of Gaussian and Cauchy kernel based MC framework was realized. The non Gaussian noise is modelled as a Gaussian mixture (glint noise) plus shot noise. Finally, the performance of the estimators were evaluated and a comparative analysis is presented on the basis of RMSE in position and % track loss. From the comparative analysis, it can be concluded that the Gaussian and Cauchy kernel based MC framework provides improved estimation accuracy than UKF and NSKF in non Gaussian noise environments. Thus, it can be inferred that MC based estimators have the potential to give accurate and robust state estimates in the presence of non Gaussian noises in angle measurements. As a future work, the proposed estimation framework can be extended to track a manoeuvring target in the presence of angles-only measurements corrupted with non Gaussian noise.

## Figures and Tables

**Table 1:** Tracking parameters for 2D scenario.

Parameters	Values
Initial Target Position	$\left[\begin{array}{cc} 4.9286 & \;0.8420 \end{array}\right]$ (km)
Initial Observer Position	$\left[\begin{array}{cc} 0 & \;0 \end{array}\right]$ (km)
Initial Target Speed (*s*)	4 (knots)
Initial Observer Speed	5 (knots)
Target Course	$-135.4^{\circ }$
Observer manoeuvre	From 780 to 1020 (s)
Initial Range (*r*)	5 (km)
Observation time	1800 (s)
$q_{x}$, $q_{y}$	9 $\times 10^{-12}$ (${\mathrm{km}}^{2}/s^{3}$)
$\sigma_{\beta }$	$1.5^{\circ }$
$\sigma_{r}$	2 (km)
$\sigma_{s}$	2 (knots)
Sampling time	T=10 (s)
Initial Observer Course	$140^{\circ }$
Final Observer Course	$20^{\circ }$
$\sigma_{c}$	$\pi /\sqrt{12}$

**Table 2:** Target & Observer Initial Parameters.

Parameters	Values
Initial Target Position	$\left[\begin{array}{ccc} 138/\sqrt{2} & \;138/\sqrt{2} & \;9 \end{array}\right]$ (km)
Initial Observer Position	$\left[\begin{array}{ccc} 0 & \;0 & \;10 \end{array}\right]$ (km)
Initial Target Speed (s)	0.297 (km/s)
Initial Observer Speed (s)	0.297 (km/s)
Target Course	$-135^{\circ }$
Observer manoeuvre	From 70 to 370 (s)
Initial Range (*r*)	150 (km)
Observation time	420 (s)
$q_{x}$, $q_{y}$	$10^{-8}\;{\mathrm{km}}^{2}/s^{3}$
$q_{z}$	$10^{-10}\;{\mathrm{km}}^{2}/s^{3}$
$\sigma_{\beta }$, $\sigma_{\epsilon }$	$0.057^{\circ }$
$\sigma_{r}$	13.6 (km)
$\sigma_{s}$	41.6 (m/s)
Elevation Angle	$0.415^{\circ }$
Sampling time	T=10 (s)

**Table 3:** 2D: RMSE in position and % Track Loss.

Filters	% Track Loss	RMSE (m)
UKF	4.4	152.8
MC-UKF-GK	1.1	111.0
MC-UKF-CK	1.1	108.9
NSKF	2.8	151.1
MC-NSKF-GK	1.2	109.6
MC-NSKF-CK	0.5	108.8

**Table 4:** 3D: RMSE in position and % Track Loss.

Filters	% Track Loss	RMSE (m)
UKF	100	-
MC-UKF-GK	14	496.1
MC-UKF-CK	13.5	499.8
NSKF	100	-
MC-NSKF-GK	14	496.8
MC-NSKF-CK	13.5	498.9

## Data Availability

Not applicable.

## Abbreviations

The following abbreviations are used in this manuscript:

$X_{k}^{t}$	Target state vector at sample *k*
${X^{o}}_{k}$	Observer state vector at sample *k*
$X_{k}$	Relative state vector at sample *k*
w	Zero mean Gaussian process noise
Q	Process covariance
F	State transition matrix
*T*	Sampling time
$q_{x},q_{y},q_{z}$	Power spectral densities of the process noise along the *X*, *Y*, and *Z* axes
$z_{k}$	Measurement vector at sample *k*
$v_{k}$	Non Gaussian measurement noise at sample *k*
r	Range vector
β and ϵ	Bearing and Elevation angle measurement
$R_{k}$	Measurement noise covariance matrix at sample *k*
$\sigma_{\beta }$ and $\sigma_{\epsilon }$	Standard deviations of error in bearing and elevation angles
$k_{\sigma }$	Kernel function
$G_{\sigma }$ and σ	Gaussian kernel and Gaussian bandwidth
$C_{\delta }$ and δ	Cauchy kernel and Cauchy bandwidth
$X_{k|k-1}$	Prior mean at sample *k*
$P_{k|k-1}$	Prior covariance at sample *k*
$W_{k}$	Weights at sample *k*
${\bar{H}}_{k}$	Measurement slope matrix
$P_{Xz}$	Cross covariance
$P_{zz}$	Measurement covariance
J	Cost function
*℘* and ϱ	Adjustable weights
$L_{i}^{G}$	Gaussian scalar term
$K_{i}^{G}$	Gaussian Kalman gain
$X_{k|k}$	Posterior mean at sample *k*
$P_{k|k}$	Posterior covariance at sample *k*
${\bar{c}}_{r}$	Initial course estimate
${\hat{\beta }}_{1}$ and ${\hat{\epsilon }}_{1}$	True initial bearing and elevation measurement estimate
${\bar{\alpha }}_{1}$ and ${\bar{\gamma }}_{1}$	Bearing and Elevation angle heading
$T_{b}$	Threshold
RMSE	Root Mean Square Error
MMSE	Minimum Mean Square Error
MC-UKF-GK	Maximum correntropy unscented Kalman filter Gaussian kernel
MC-UKF-CK	Maximum correntropy unscented Kalman filter Cauchy kernel
MC-NSKF-GK	Maximum correntropy new sigma point Kalman filter Gaussian kernel
MC-NSKF-CK	Maximum correntropy new sigma point Kalman filter Cauchy kernel

## Appendix A. Power Series Expansion of Cauchy Kernel Function

The binomial expansion of ${\left(1+x\right)}^{-N}$ for negative integer −N is given as follows:

1+x−N=1+(−N)x+(−N)(−N−1)2!x2+(−N)(−N−1)(−N−2)3!x3+⋯,=∑k=0∞(−1)kN+k−1kxk,for|x|<1.

Now the correntropy measure, by taking the binomial series expansion of Cauchy kernel with $x=\frac{{\left(X-Y\right)}^{2}}{\delta }$ is

Vδ(X,Y)=∑k=0∞(−1)kN+k−1k(X−Y)2δk=∑k=0∞(−1)kδkN+k−1kE(X−Y)2k,for|(X−Y)2δ|<1.

## Appendix B. Derivation of Kalman Gain

The Kalman gain for Gaussian kernel based nonlinear estimator is $K_{k}^{G}$ with $L_{k}^{G}$ as a scalar term. Similarly, for Cauchy kernel, it is $K_{k}^{C}$ and $L_{k}^{C}$ respectively. A general expression for Kalman gain is given as $K_{k}={\left(P_{k|k-1}^{-1}+L_{k}{\bar{H}}^{\prime }R_{k}^{-1}\bar{H}\right)}^{-1}L_{k}{\bar{H}}^{\prime }R_{k}^{-1}$. Applying matrix inversion lemma

$$\begin{array}{cc} K_{k} & =\left(P_{k|k-1}-P_{k|k-1}L_{k}{\bar{H}}^{\prime }{\left(R_{k}+\bar{H}P_{k|k-1}L_{k}{\bar{H}}^{\prime }\right)}^{-1}\bar{H}P_{k|k-1}\right)L_{k}{\bar{H}}^{\prime }R_{k}^{-1}\\ \; & =P_{k|k-1}L_{k}{\bar{H}}^{\prime }R_{k}^{-1}-P_{k|k-1}L_{k}{\bar{H}}^{\prime }{\left(R_{k}+\bar{H}P_{k|k-1}L_{k}{\bar{H}}^{\prime }\right)}^{-1}\bar{H}P_{k|k-1}L_{k}{\bar{H}}^{\prime }R_{k}^{-1}\\ \; & =P_{k|k-1}L_{k}{\bar{H}}^{\prime }\left(R_{k}^{-1}-{\left(R_{k}+\bar{H}P_{k|k-1}L_{k}{\bar{H}}^{\prime }\right)}^{-1}\bar{H}P_{k|k-1}L_{k}{\bar{H}}^{\prime }R_{k}^{-1}\right)\\ \; & =P_{k|k-1}L_{k}{\bar{H}}^{\prime }\left(I-{\left(R_{k}+\bar{H}P_{k|k-1}L_{k}{\bar{H}}^{\prime }\right)}^{-1}\bar{H}P_{k|k-1}L_{k}{\bar{H}}^{\prime }\right)R_{k}^{-1}. \end{array}$$

After certain algebraic manipulations, we get

(A1) $$K_{k}=P_{k|k-1}L_{k}{\bar{H}}^{\prime }{\left(I+R_{k}^{-1}\bar{H}P_{k|k-1}L_{k}{\bar{H}}^{\prime }\right)}^{-1}R_{k}^{-1}=P_{k|k-1}L_{k}{\bar{H}}_{k}^{\prime }{\left(R_{k}+{\bar{H}}_{k}P_{k|k-1}L_{k}{\bar{H}}_{k}^{\prime }\right)}^{-1}.$$

From the above equation, $K_{k}^{G}$ and $K_{k}^{C}$ can be defined by making necessary substitution for $L_{k}^{G}$ and $L_{k}^{C}$.
